# Evidences and drivers of ocean deoxygenation off Peru over recent past decades

**DOI:** 10.1038/s41598-021-99876-8

**Published:** 2021-10-13

**Authors:** D. Espinoza-Morriberón, V. Echevin, D. Gutiérrez, J. Tam, M. Graco, J. Ledesma, F. Colas

**Affiliations:** 1 Instituto del Mar del Peru (IMARPE), Esquina General Gamarra y Valle, Callao, Peru; 2 LOCEAN-IPSL, IRD/Sorbonne Université/CNRS/MNHN, Paris, France; 3 Programa de Maestría en Ciencias del Mar, Universidad Peruana Cayetano Heredia, Lima, Peru

**Keywords:** Biogeochemistry, Environmental sciences, Ocean sciences

## Abstract

Deoxygenation is a major threat to the coastal ocean health as it impacts marine life and key biogeochemical cycles. Understanding its drivers is crucial in the thriving and highly exploited Peru upwelling system, where naturally low-oxygenated subsurface waters form the so-called oxygen minimum zone (OMZ), and a slight vertical shift in its upper limit may have a huge impact. Here we investigate the long-term deoxygenation trends in the upper part of the nearshore OMZ off Peru over the period 1970–2008. We use a unique set of dissolved oxygen in situ observations and several high-resolution regional dynamical-biogeochemical coupled model simulations. Both observation and model present a nearshore deoxygenation above 150 m depth, with a maximum trend of – 10 µmol kg^−1^ decade^1^, and a shoaling of the oxycline depth (− 6.4 m decade^−1^). Model sensitivity analysis shows that the modeled oxycline depth presents a non-significant (+ 0.9 m decade^−1^) trend when remote forcing is suppressed, while a significant oxycline shoaling (− 3 m decade^−1^) is obtained when the wind variability is suppressed. This indicates that the nearshore deoxygenation can be attributed to the slowdown of the near-equatorial eastward currents, which transport oxygen-rich waters towards the Peruvian shores. The large uncertainties in the estimation of this ventilation flux and the consequences for more recent and future deoxygenation trends are discussed.

## Introduction

Deoxygenation is a major threat to the health of the ocean’s ecosystems. During the last decades, progressive deoxygenation was evidenced in the upper and deep-open ocean^1,2^, and in several coastal regions^3^. In the open ocean, warming since the middle of twentieth century induced a decrease of dissolved oxygen (DO) solubility, an intensification of the stratification, and likely changes in the DO consumption rates by marine biota, which together have produced a DO loss^2,4^. These processes may also play a role in the deoxygenation of coastal waters, which may be aggravated by human-induced coastal eutrophication^3^.

Deoxygenation is likely to persist for decades onward. Indeed, most of the Earth System Models (ESM) project a persistence of DO loss to the atmosphere and a reduction of 3,5% of total DO in 2090 respect to 1990 under the worst-case climate change scenario (RCP8.5)^5^. However, these models tend to underestimate the observed deoxygenation over the historical period (1960–2018)^4^.

**Figure 1:**
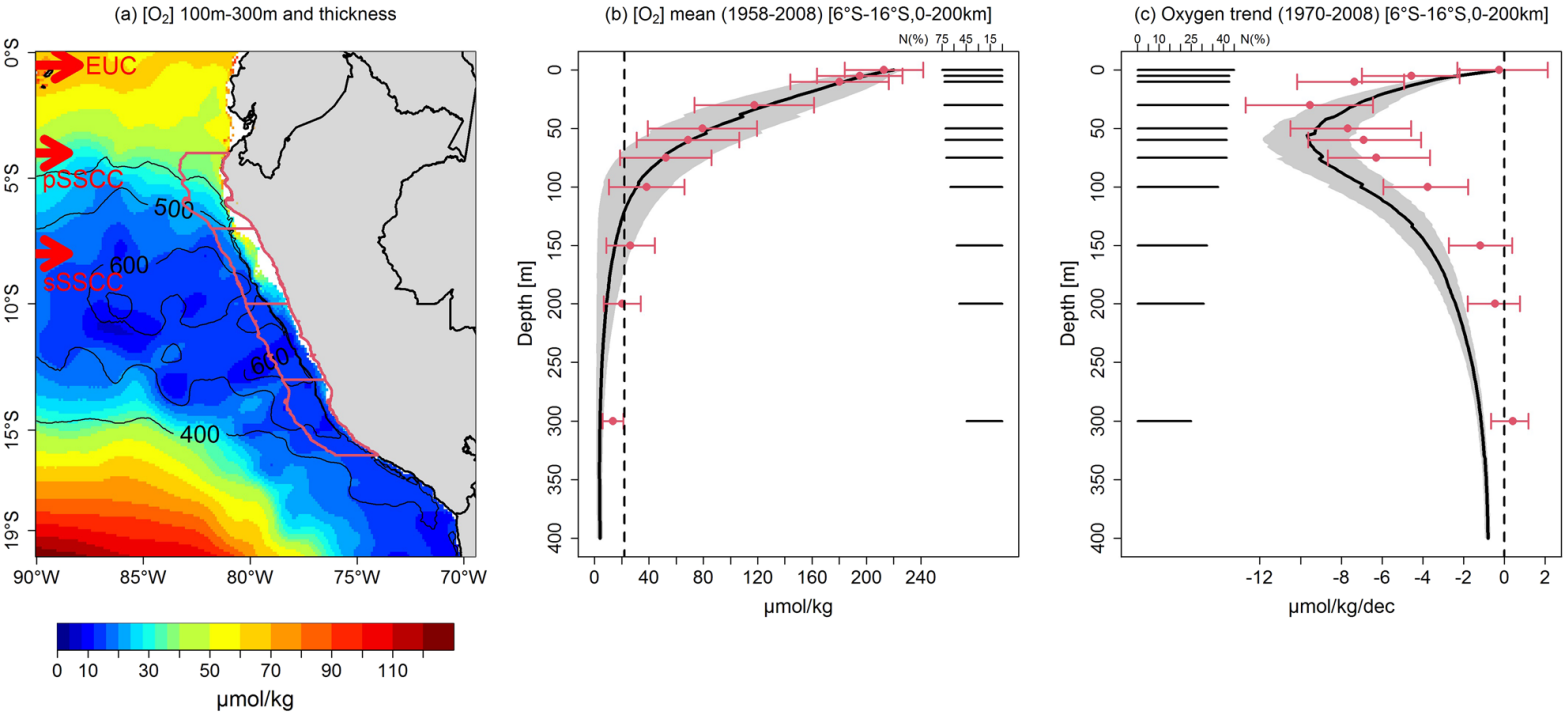
Dissolved oxygen mean state and trends off Peru. (**a**) Mean observed DO concentration (in µmol kg^−1^; color shading) and OMZ thickness (in meters; black contours); (**b**) mean observed (red dots) and modeled (black line) DO (in µmol kg^−1^) in a coastal band of 200 km, between 6° and 16° S; (**c**) DO linear trend (in µmol kg^−1^ dec^−1^) computed over 1970–2008 from IMARPE data (red dots) and model output (black line) at different depth levels. Black horizontal bars indicate the percentage of months with observations with respect to the total number of months (468) in the 1970–2008 period for each depth (right side of (**b**) and left side of (**c**)). Red bars and grey shading in (**b**) and (**c**) indicate error bars for the observed and model values, respectively. The figures were generated using the software R (R Core Team, version 4.1.1., http://www.R-project.org/).

Eastern boundary upwelling systems (e.g. the California, Humboldt, Canary and Benguela Upwelling Systems) encompass subsurface body waters (usually located above 1000 m depths) with low DO concentration, called Oxygen Minimum Zones (OMZs, defined in this study as [O_2_] < 22 µmol kg^−1^)^6^. The northern Humboldt Current System^7^, also called the Peruvian Coastal Upwelling System (PCUS; 4° S–18° S) overlaps one of the most intense and shallow OMZs^8,9^ (Fig. 1a,b), where anoxic episodes can occur^10^. Complex physical and biogeochemical processes produce the formation and maintenance of this regional OMZ. Located in the poorly ventilated “shadow zone” of the Tropical South Eastern Pacific^11^, OMZ waters have a long residence time^12^, and are subject to high oxygen consumption due to the remineralization of a large amount of organic matter^6^. The OMZ is also shaped by mesoscale eddies and filaments^9,13^ and is ventilated on its western flank by the eastward oxygen-rich subsurface zonal currents, i.e. the Equatorial Undercurrent (EUC) and the primary (pSSCC) and secondary (sSSCC) Subsurface Counter currents^12,14^. The OMZ interannual variability is mainly forced by the El Niño Southern Oscillation (ENSO)^9,10^.

The OMZ variability at multidecadal time scales is less known. The equatorial part of the OMZ is supposed to be expanding vertically since the 1960s^2,15,16^. In the nearshore PCUS, the depth of the oxycline (defined here by the 22 μmol kg^−1^ iso-surface located on the upper part of the OMZ; Fig. S1) is often used as a proxy of near-surface layer (0–100 m) oxygen depletion. The PCUS oxycline undergoes a shoaling since the 1980s until the 2010s^17^, likely associated to a large-scale climate oscillation, the Pacific Decadal Oscillation (PDO)^18^. At greater depths (> 150 m) and closer to the OMZ core, a moderate oxygenation trend was evidenced between 1960 and 2010, attributed to an increased ventilation by the eastward equatorial undercurrents^19^ or by a deep equatorward coastal current^20^ transporting more oxygenated intermediate waters.

In this work, we study the PCUS DO trends and drivers over the period 1970–2008. We make use of (i) a comprehensive dataset of in situ DO measurements and (ii) mesoscale-resolving regional coupled physical-biogeochemical model simulations. The model reproduces clearly the observed DO trends in the upper water column, characterized by a marked shoaling of the oxycline, which allows to unravel its main drivers.

## Results

### Evidences of deoxygenation

We first characterize the mean state of the PCUS OMZ over the 1958–2008 period. Between 100 and 300 m depth, hypoxic waters (< 30 µmol kg^−1^) are encountered between the coast and as far offshore as 90°W off Central Peru (Fig. 1a). The OMZ thickness (defined by the distance between the upper and lower 22 µmol kg^−1^ iso-surfaces) reaches ~ 600 m nearshore between 10° S and 15° S. Nearshore observed DO values decreases from surface (~ 240 µmol kg^−1^) to bottom (i.e. ~ 10 µmol kg^−1^ at 300 m). This structure is well reproduced by the model although DO values are slightly weaker (~ 5–10 µmol kg^−1^) below 150 m depth (Fig. 1b). This discrepancy could be partly related to the IMARPE data set, which includes Nansen and Niskin bottle measurements known to be insufficiently accurate at very low DO values^9^.

A marked deoxygenation between 10 and 100 m depth is evidenced nearshore over the 1970–2008 time period (Figs. 1c, S1, S2). Observations and model show a maximum deoxygenation trend of ~  − 10 µmol kg^−1^ dec^−1^ at 30 and 50 m depth, respectively, corresponding to a DO loss of 40 µmol kg^−1^ in 40 years. However, model trends are stronger between 50 and 300 m depth.

**Figure 2:**
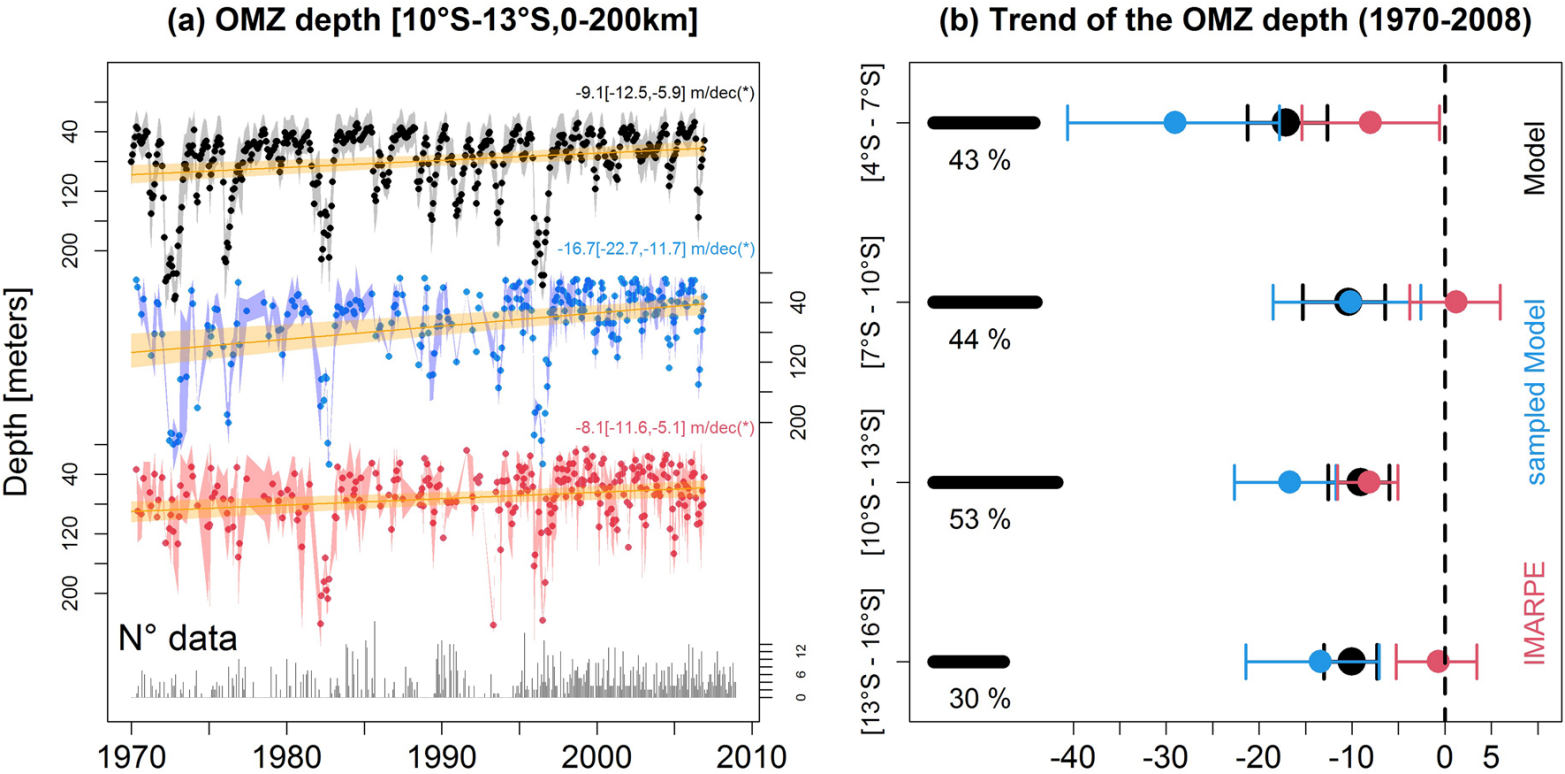
Oxycline trends along the Peruvian coasts. (**a**) Oxycline depth (in meters) over 1970–2008, averaged in a coastal box (10–13° S, from the coast to 200 km offshore) from IMARPE observations (red), model (black) and model output subsampled in the same way as the observations (blue). The number of observations per month is indicated at the bottom of the figure. Linear trend values are marked by orange line. Trend values and confidence interval are indicated on the right of the figure. Asterisk denotes a statistically significant trend. (**b**) Oxycline depth trend for different coastal bands: 4–7° S, 7–10° S, 10–13° S, 13–16° S, from IMARPE observations (red dots), model (black dots) and subsampled model (blue dots). The dashed vertical line delimits negative trends. The percentage of months with observations is indicated on the left side of (**b**). Error bars (horizontal segments) are computed using a bootstrap method. The figures were generated using the software R (R Core Team, version 4.1.1., http://www.R-project.org/).

In the following, we focus on the upper part of the OMZ and characterize the nearshore oxycline trends and their alongshore variability. The nearshore oxycline (averaged between 10° S and 13° S) varies strongly at different time scales (Fig. 2a). A marked deepening (~ 100 m) associated to the passage of intense ‘downwelling’ coastal trapped waves during the strong 1982–1983 and 1997–1998 El Niño events can be seen^9^. The nearshore oxycline progressively shoals over the 40-year time period. Note that subsampling the model DO in the exact same way as the observations leads to a stronger shoaling (− 16.7 m dec^−1^ vs − 9.1 m dec^−1^).

The oxycline trends clearly depend on latitude (Fig. 2b). Significant observed shoaling trends (~ − 8 m dec^−1^) are found only in the north (4° S–7° S) and off central Peru (10° S–13° S). In contrast, the model simulates significant shoaling trends all along the coast, whose intensity decreases poleward. Again, stronger trends are found (blue lines in Fig. 2) when model output is subsampled. This suggests that the model tends to overestimate the magnitude and extent of the deoxygenation, and also that the observed trends may be overestimated with respect to the ‘real’ trends due to the inhomogeneous spatio-temporal observational sampling.

### Deoxygenation drivers

**Figure 3:**
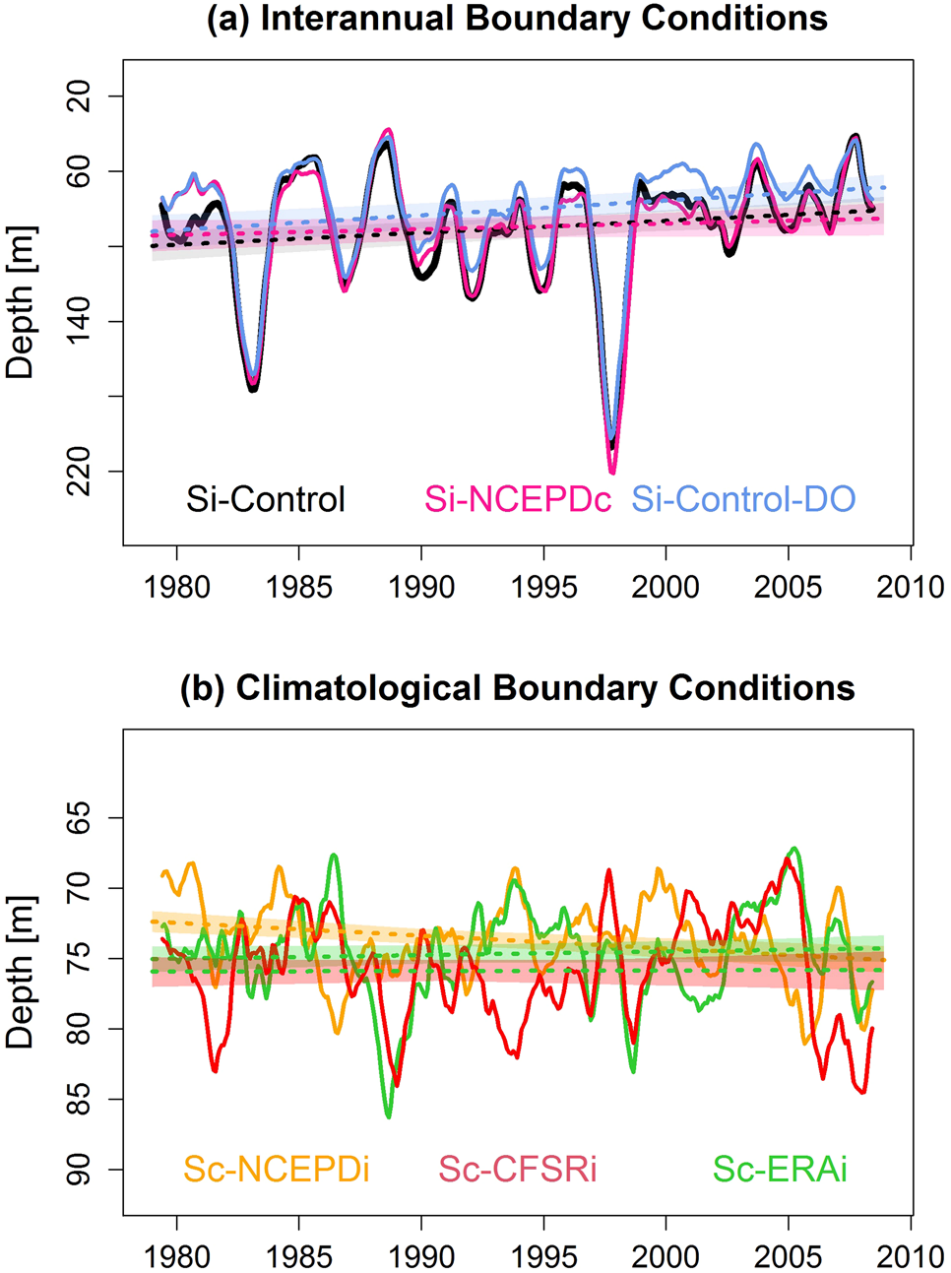
Oxycline response to remote and local forcing. Temporal evolution of modeled oxycline depth for the model experiments described in Table 1. Model simulations are either forced by interannual boundary conditions (Si-Control (black line), Si-NCEPDc (cyan line), Si-Control-DO (blue line), or by climatological boundary conditions and interannual wind forcing (Sc-NCEPDi (yellow line), Sc-CFSRi (red line), Sc-ERAi (green, line). Note the change of scale in the vertical axis in (**a**) and (**b**) marking the weaker trends in (**b**). The figures were generated using the software R (R Core Team, version 4.1.1., http://www.R-project.org/).

In order to attribute the nearshore deoxygenation to remote or local forcing, the modeled oxycline trends are then computed for simulations forced by different atmospheric and oceanic conditions (Table 1). A marked shoaling trend is obtained when near-equatorial remote oceanic variability is allowed to propagate into the PCUS through the model open boundaries (Fig. 3a). The trend is weakly impacted by the different interannual atmospheric forcings, but reduces by half when interannual variability of the wind is suppressed (Table 1), showing that wind variability enhances deoxygenation. Moreover, the trend increases by 20% when a subsurface deoxygenation trend derived from observations^21^ is introduced in the offshore equatorial region (Table 1, Fig. 3a). In contrast, when the remote equatorial variability is suppressed, the trends are much weaker than the observations (Fig. 3b). Only NCEP wind forcing drives a weak (+ 0.9 m dec^−1^) statistically significant oxycline deepening, contrasting with the observed trend (Table 1).

**Table 1:** Numerical simulations design.

Simulation name	Time period	Wind forcing	Physical OBC	Biogeochemical OBC	DO OBC	Oxycline depth trend (m dec^−1^)
Si-Control	1958–2008	NCEP downscaled int	SODA int	CARS-WOA clim	CARS clim	− **6.4 [**− **9.0,** − **3.7]**
Si-CFSRi	1979–2008	CFSR* int	SODA int	CARS-WOA clim	CARS clim	− **4.1 [**− **6.6,** − **1.6]**
Si-ERAi	1979–2008	ERAI* int	SODA int	CARS-WOA clim	CARS clim	− **4.3 [**− **7.0,** − **1.7]**
Si-Control-DO	1979–2008	NCEP downscaled int	SODA int	CARS-WOA clim	CARS clim. + trend	− **7.8 [**− **10.5,** − **5.2]**
Si-NCEPDc	1979–2008	NCEP downscaled clim	SODA int	CARS-WOA clim	CARS clim	− **3.0 [**− **6.1,** − **0.2]**
Si-SCOWc	1979–2008	SCOW clim	SODA int	CARS-WOA clim	CARS clim	− **3.0 [**− **5.9,** − **0.1]**
Sc-NCEPDi	1979–2008	NCEP downscaled int	SODA clim	CARS-WOA clim	CARS clim	**0.9 [0.6,1.2]**
Sc-CFSRi	1979–2008	CFSR* int	SODA clim	CARS-WOA clim	CARS clim	− 0.03 [− 0.5, 0.4]
Sc-ERAic	1979–2008	ERAI* int	SODA clim	CARS-WOA clim	CARS clim	0.25 [− 0.6, 0.06]

Characteristics of the numerical simulations (simulation name, time period, wind forcing, physical open boundary conditions (OBC), biogeochemical OBC, DO OBC and oxycline trend). Asterisks in the wind forcing column indicate that the forcing is composed of a SCOW monthly climatology and atmospheric model monthly anomalies. “int” means interannual and “clim” means climatological. Oxycline trends are indicated in bold font when statistically significant, along with the corresponding confidence interval.

Near-surface nearshore deoxygenation thus appears to be mainly driven by the remote equatorial forcing variability associated with the Equatorial undercurrent and subequatorial eastward jets (Fig. S3). The EUC, pSSCC and sSSCC mass and DO fluxes exhibit decreasing trends at 88°W, west of the PCUS (Table 2). Such a reduced input of oxygen into the PCUS is likely to have contributed to the deoxygenation. Statistically significant correlations between the annual eastward oxygen flux and the annual nearshore oxycline depth are also found in various latitude bands, the strongest correlation being in the north (6° S–8° S, Table S1). The stronger correlations between the oxycline and SSCCs at interannual time scales suggest that the SSCCs have a stronger influence on the nearshore OMZ than that of the EUC, in agreement with previous studies^22,23^.

**Table 2:** EUC and SSCC mass and oxygen flux trends over 1970–2008.

	Flux (Sv dec^−1^)		Oxygen (µmol kg^−1^ dec^−1^)		Oxygen flux (10^3^ mol s^−1^ dec^−1^)	
	Trend	Conf. Int	Trend	Conf. Int	Trend	Conf. Int
EUC	− 0.36	[− 0.51 to − 0.21]	− 1.62	[− 2.21 to − 1.05]	− 52.21	[− 74.39 to − 30.64]
pSSCC	− 0.37	[− 0.44 to − 0.31]	− 3.49	[− 4.47 to − 2.52]	− 49.76	[− 60.19 to − 39.82]
sSSCC	− 0.14	[− 0.21 to − 0.08]	− 4.36	[− 5.77 to − 3.15]	− 35.27	[− 45.23 to − 27.13]

Linear trend of the eastward mass flux (in Sv dec^−1^), oxygen concentration (in μmol kg^−1^ dec^−1^), and eastward oxygen flux (in 10^3^ mol s^−1^ dec^−1^) associated with the near-equatorial eastward jets (EUC, pSSCC, sSSCC), computed at 88° W over 1970–2008 from the control simulation (Si-Control). Eastward velocities greater than 0.2 cm s^−1^ are considered in the computation.

### Biogeochemical trends of DO

On average over the entire period, the maximum DO consumption rate (due to biogeochemical processes) near the coast was ~  − 65 μmol kg^−1^ year^−1^ at ~ 70 m (Fig. S4a), and the linear change of DO consumption between 1970 and 2008 was − 0.3 μmol kg^−1^ year^−2^, which represents a total decrease of ~ 10 μmol kg^−1^ year^−1^ (Fig. S4b). Taking into account this linear change, biogeochemical processes have produced ~  + 230 μmol kg^−1^ of DO over 40 years (between 1970 and 2008; see supplementary information). This needs to be compared to the net DO loss of ~ 40 μmol kg^−1^ over the same period (Fig. 1c) due to both physical and biogeochemical processes. In conclusion, nearshore deoxygenation is driven by the physical processes and partly compensated by a reducing biogeochemical DO sink.

### The role of EUC and SSCCs

**Figure 4:**
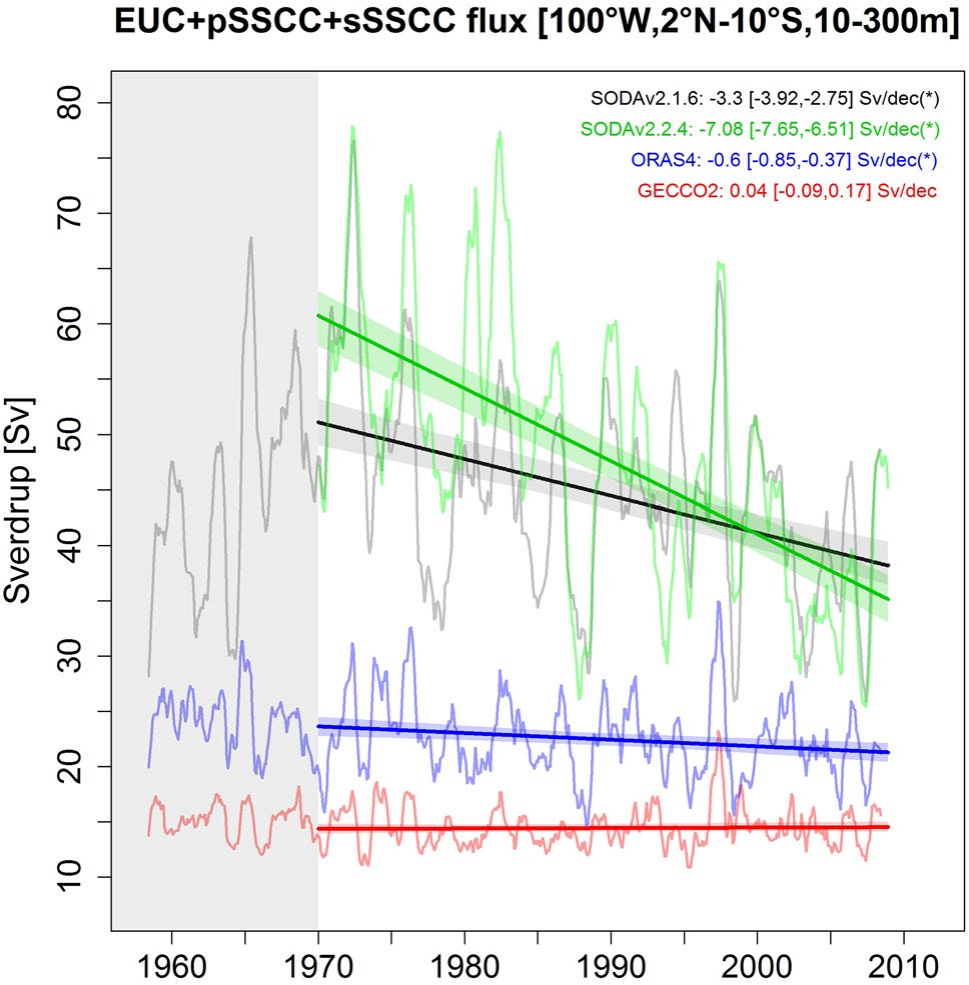
Near-equatorial eastward mass flux trends over 1970–2008. Eastward flux (in Sv) at 100° W associated with the near-equatorial eastward undercurrents (EUC, pSSCC, sSSCC) for 4 different reanalysis global products over 1958–2008: SODAv2.1.6 (black), SODAv2.2.4 (green), ORAS4 (blue), GECCO2 (red). Linear trends over 1970–2008 and confidence intervals are indicated in the upper right side of the figure and asterisks indicate statistical significance. The figure was generated using the software R (R Core Team, version 4.1.1., http://www.R-project.org/).

As near-equatorial jets seem to play a major role in the deoxygenation trends, we now examine the eastward mass flux at 100° W (the model western boundary) in a near equatorial band including the EUC and SSCCs (2° N–10° S) in four different ocean reanalyses (Figs. 4, S5). The SODAv2.1.6 reanalysis (forcing our model) simulates a much stronger mean eastward flux (~ 40–60 Sv) than ORAS4 (~ 20–25 Sv) and GECCO2 (~ 15 Sv) over the period. Furthermore, the asynchronous interannual flux variations in the reanalyses illustrate the difficulty to estimate it accurately. Three out of the four reanalyses simulate a flux decrease with a weaker downtrend than SODAv2.1.6 for two of them (Fig. 4). This suggests that the simulated eastward mass and DO fluxes may decrease too strongly and induce an overly strong deoxygenation. It possibly explains the overestimated modeled trend compared to the observed (Fig. 2).

## Discussion

### Physical versus biogeochemical processes

Biogeochemical processes play a complex role in the deoxygenation of subsurface coastal waters. Indeed, the progressively shoaling oxycline driven by the circulation changes is associated with a nutricline shoaling (figure not shown), which may bring more nutrient to the surface layer, enhance primary productivity, organic matter (OM) export and remineralization in the subsurface layer, amplifying the deoxygenation^24^. However, the DO consumption by biogeochemical processes in our model decreased over the period of study. This decrease can be explained as follows: as the ventilation of the upper OMZ decreases and the oxycline shoals, the proportion of OM exported in the low oxygen part of the water column increases. As there is not enough oxygen to support OM aerobic remineralization, anaerobic processes like denitrification/anammox take over^25^. This process was also evidenced during La Niña events^9,26^.

### Comparison with previous studies

Our results imply that deoxygenation in the PCUS between the 1970s and the 2000s is closely linked to a decreasing DO eastward flux offshore of the OMZ. The EUC and SSCCs have been previously identified as strong drivers of OMZ variability as they transport equatorial waters into the OMZ, which are less oxygen-depleted than the coastal waters^9,14,27–29^. In a low-resolution (2°) modeling study, Duteil et al.^30^ attributed to a large-scale circulation slow-down the volume expansion (+ 7%) of the volume of suboxic regions (< 5 μmol kg^−1^) in the Eastern Pacific Ocean during a PDO positive phase. The decreasing trade winds in the Eastern Tropical Pacific associated with the mostly positive PDO phase during 1979–2008 may be responsible for decreasing the near-equatorial eastward ventilation flux^31^ but also for decreasing the equatorial waters oxygen content in the Eastern equatorial Pacific (east of ~ 140° W) through a reduction of subtropical thermocline ventilation.

Other studies reported observed oxygen trends different from our findings, depending on the time period and depth range considered. A weak deoxygenation trend was found over 1960–2017^15^, albeit not significant because of strong trends reversal related to the different PDO phases of the period. Proxy and instrumental records inferred an oxygenation trend in the core of the OMZ (100–300 m depth) over the Peruvian central margin from 1865 to 2004^19^. To explain this trend, an eastward velocity increase in the EUC core, over the period 1860–2008 near 150° W, was inferred from an ocean reanalysis (SODA). However, the SODA velocity increase was strong in the central equatorial Pacific and weaker eastward, reaching nearly zero near 90° W^32^. An alternative explanation for the OMZ core oxygenation is a possible stronger ventilation over the margin driven by the equatorward transport of deep oxygenated Intermediate Circulation Subantarctic waters (ICSAW)^19,20^. It is remarkable that a modelling study^9^ obtained less oxygen content in the water column in the absence of ICSAW than when it is present during La Niña, but this was not further explored. This implies that more investigations are needed to better understand the processes inducing oxygenation in the core and lower part of the OMZ.

### DO trends in the last two decades

An oxygenation trend associated with a deepening of the oxycline (− 0.64 m decade^−1^) has been observed at a fixed point over the central Peruvian shelf (12° S, 0–150 m depth range) over a more recent period (1999–2011)^10^. To investigate the potential role of the eastward near-equatorial circulation over this time period, we computed the eastward transport linear trends at 100° W using 5 recent global ocean reanalyses (Fig. S6, Table S2). The significant negative trends obtained for three out of the five reanalyses argue for a decreasing eastward flux over this period. Thus, the oxygenation trend over 1999–2011 contrasts with the possible slow-down of the near-equatorial eastward circulation evidenced in the reanalyses. However, as shown in one model experiment (Si-Control-DO, Table 1), in which a DO decrease of the water masses was artificially included at 100°W, in agreement with in situ observations^21^ (Fig. S5a), the DO evolution of subsurface equatorial waters also impacts the DO trends (Fig. 3a). The recent strengthening of the wind-driven Subtropical-Tropical Cells, which transport oxygen from the subtropics to the Tropical Pacific^33^, may induce a reoxygenation of Eastern Pacific equatorial waters. A stabilization of DO levels has been observed in the eastern equatorial Pacific (110° W) from 2000 to 2010 after a period of stronger decrease^34^. This effect may thus compensate the impact of the decreasing eastward water flux over 1999–2011. Note also that local trends (e.g. at 12° S^10^) can be heterogeneous alongshore^35^ and cross-shore (Fig. S1) and may not be representative of broader scales. To conclude, note that all the reanalysis products present a positive trend over the most recent period (2005–2017, Table S2); however, only two models are statistically significant, which suggests a probable regime shift of the oxygenation during the last 10–15 years.

### Drivers of near-equatorial eastward flux and DO variability

Another striking result of our study is the strong variability of the eastward volume flux trends (at 100° W) among the ocean reanalyses that we analysed. The trade winds, which are part of the low-level branch of the Walker circulation (hereafter WC), are supposed to be the main forcing of the EUC: as the wind-driven surface currents push water westward, the associated sea level rise generates a zonal pressure gradient that drives the EUC across the equatorial Pacific. Observational studies indicate that the WC has intensified over the recent decades^36^, in association with an increased zonal Sea Surface Temperature (SST) gradient across the Pacific, which is at odds with the EUC decrease in SODA (Table 2). Furthermore, the very different eastward flux trends in two ocean reanalyses (SODAv2.1.6 and ORAS4) forced with the same surface winds (ERA-40) show that wind variability may not be the main driver of the trend.

Another possible driver of DO variability in the PCUS is the long-term evolution of eddy variability. The impact of this physical forcing on the OMZ long-term trends has not been investigated in the present study, although eddies and filaments are resolved in our 1/6° regional model. These processes are known to impact the OMZ variability at seasonal^13^ and interannual scales^9^. The decrease of the eastward flux may impact the PCUC flux and vertical structure and thus modulate eddy kinetic energy at long time scales. However, the high correlation levels between the eastward flux and oxycline suggest that these effects may be of second order. A dedicated study would be needed to evaluate the impact of eddy variability on the OMZ trends.

### Potential sources of model-data discrepancies

A lack of horizontal resolution in ocean global circulation models (OGCMs) may lead to an unrealistic eastward shoaling of the oxycline, and an exaggerated sensitivity to EUC changes^28^. Discrepancies in the eastward flux derived from the reanalyses could be attributed to the different OGCMs physical parameterizations, data assimilation methods and observations used in the data assimilation process. Consequently, the stronger shoaling of the oxycline depth in our regional model with respect to observations could be partly produced to a biased representation of the eastward circulation in the near equatorial band in the SODA reanalysis. On the other hand, the deoxygenation of eastern equatorial Pacific waters over 1960–2008^2,15^, poorly simulated by OGCMs^4^, may intensify the nearshore deoxygenation associated with the near-equatorial ventilating flux slow-down (see the Si-Control-DO experiment, Table 1). Another limitation of our modelling approach is the use of DO climatological boundary conditions. Global ocean reanalysis including biogeochemistry are highly needed as a coherent physical-biogeochemical variability needs to be taken into account in regional model open boundary forcing.

### DO long-term variability in other EBUS

Other EBUS regions may be influenced by near-equatorial long-term variability. In the Eastern Pacific Ocean, the OMZ off north and central Chile is mainly modulated by the PCUC poleward transport of oxygen at seasonal timescales^37^. As the PCUC is strongly connected to the EUC and SSCCs^9,22,23^, a weaker eastward transport may induce a weaker poleward flux of low oxygenated waters along the Chilean slope and an oxygenation trend (e.g. at 36° S)^38^, assuming that advective processes are dominant at these time scales. Long-term deoxygenation has also been reported in the north Eastern Pacific, in particular in the southern California Current System. A shoaling of the hypoxic boundary (60 μmol L^−1^) of ~ 45 m decade^−1^ over the period 1984–2006 was attributed to the transport of low-DO subtropical waters by the poleward California Current^39^. Positive PDO phases induced a deoxygenation off the coasts of Mexico mainly due to advective processes^30^, while PDO-associated variability propagated along the coasts of central and north America triggering a heaving of isopycnals and oxygen isosurfaces^16,24,40^. However, how much of this variability is related to the near-equatorial eastward ventilating flux remains to be quantified.

Recently, Brandt et al.^41^ related the oxygenation of subsurface waters in the Eastern Equatorial Atlantic ocean to an increase of eastward transport of the equatorial undercurrent between 2008 and 2018. Unlike ours, their study was based on current and dissolved oxygen observations only. Even though this oxygenation trend occurred in a different tropical ocean, the dynamical processes involved are rather similar, which supports our conclusions.

## Conclusions

In-depth analysis of in situ observations and model suggest that the progressive deoxygenation of the upper part of the OMZ off Peru over 1979–2008, as reflected by the shoaling of the oxycline, is driven by a reduction of the eastward transport of the less oxygen-depleted waters from the near equatorial band to the South Eastern Tropical Pacific OMZ. The nearshore deoxygenation could be intensified in the case of a decrease of the tropical waters DO content. Future work is needed to better understand the mechanisms driving the long-term variability of the ventilating flux, and to disentangle the physical processes driving the EUC and SSCCs volume flux variability from the processes modulating the DO content of tropical waters.

More studies are also needed to investigate in more detail the ventilating role of the intermediate waters equatorward advection on the OMZ core and lower layers, as well as of the eddy variability at decadal time scales. New in situ observations in the tropical south Eastern Pacific Ocean as well as consistent biophysical global reanalyses will be needed to tackle these questions.

## Material and methods

### IMARPE dissolved oxygen data

Thanks to a sustained observational effort carried out by the Peruvian Marine Institute (Instituto del Mar del Peru, IMARPE) over more than 50 years, approximately ∼15,000 DO vertical profiles were collected between 1961 and 2008^17^. Most of the observations (96%) used in the present study are from Nansen and Niskin bottles that were collected during regular surveys and at fixed stations along the coast. DO was determined by the Winkler method^42^. 5% of the dataset (~ 800 profiles) are from CTD-O casts deployed between 1981–1984 and 1991–1994. In addition to IMARPE DO observations, 1500 profiles from the World Ocean Database (WOD09) were used. The DO profiles were interpolated to 55 standard depth levels from the surface to 1000 m depth, with 37 points above 300 m^17^. Each DO profile was linearly interpolated on a vertical grid with a 1-m resolution to compute the oxycline depth. The oxycline depth values were then averaged horizontally in the coastal region to produce an index characterizing the variability of the OMZ upper limit in the nearshore region. Data availability in different latitude bands and decades are shown in Fig. S7 and summarized in Table S3.

### ROMS-PISCES regional ocean model

The Regional Oceanic Modeling System model^43^ was used to simulate the ocean dynamics. The ROMS-AGRIF code (version 3.1) was used. ROMS resolves the Primitive Equations, based on the Boussinesq approximation and hydrostatic vertical momentum balance. Wind stress, fresh water, sensible and latent heat fluxes were computed using a bulk parameterization. ROMS was coupled to the Pelagic Interaction Scheme for Carbon and Ecosystem Studies (PISCES)^44^ model to simulate marine biological productivity and the biogeochemical cycles of carbon and main nutrients (P, N, Si, Fe). PISCES has three non-living compartments (semi-labile dissolved organic matter, small sinking particles and large sinking particles) and four living compartments represented by two size classes of phytoplankton (nanophytoplankton and diatoms) and two size classes of zooplankton (microzooplankton and mesozooplankton). It also simulates the DO cycle driven by advection and subgrid mixing, air-sea exchanges (note that the O_2_ concentration in the atmosphere is assumed to be constant and spatially homogeneous over the whole time period of the simulation), production by photosynthesis and consumption by plankton respiration and organic matter remineralization.

### Model configuration

The model domain spans from 15° N to 40° S and from 100° W to 70° W. The horizontal grid of 1/6° (∼18 km) allows to represent mesoscale structures off Peru (the Rossby radius of deformation is equal to ~ 70 km). The ETOPO2 bottom topography is used, and the vertical grid has 32 sigma levels. The same model configuration has been used to study the interannual variability of productivity and oxygen associated with ENSO^9,45^.

### Open boundary conditions

5-day average open boundary conditions (OBC) for physical variables came from the Simple Ocean Data Assimilation (SODA) model solution (version 2.1.6) over the period 1958–2008. SODA EUC maximum eastward velocity is very close to that measured by the Tropical Atmosphere Ocean (TAO) acoustic Doppler current profilers (ADCP) over 1990–2008 at 110° W^32^, close to the western boundary of the regional model (100° W). Besides, a SODA monthly climatology (constructed over 1958–2008) was used as physical OBC in various sensitivity simulations (see Table 1).

Since an interannual simulation of the biogeochemical conditions in the Eastern Pacific was not available for the period of study (1958–2008), OBC from the CARS2009 climatology were used for nutrients (nitrate, silicate, phosphate) and DO. OBC from the Global Ocean Data Analysis Project (GLODAP) data base^46^ were used for dissolved organic carbon, dissolved inorganic carbon and total alkalinity. As a gridded climatology of iron measurements does not exist, iron OBC from a climatology of a NEMO-PISCES global simulation were used^44^. This simple approach for biogeochemical boundary values produced satisfactory results^9,45^. Last, a perturbed DO boundary condition was constructed in the equatorial region (100°W) by adding an oxygen trend^21^ to climatogical DO in one model sensitivity experiment (Si-Control-DO, see Table 1).

### Atmospheric forcing

Several surface wind fields were used to force the PCUS regional model (see Table 1), in order to test the sensitivity of the results to different regional wind forcings. The forcing used in the control experiment (Si-Control, Table 1) was obtained by summing statistically-downscaled NCEP daily wind anomalies^47^ and the SCOW monthly climatology. Using SCOW as mean wind state allows to correct the atmospheric reanalysis mean bias and greatly improves the realism of the circulation^48^. Similar forcing (CFSR*, ERAI*, see Table 1) were constructed by adding CFSR (the NCEP Climate Forecast System Reanalysis) and ERAI (the ECMWF Interim Reanalysis) daily wind anomalies to the SCOW monthly climatology. NCEP daily anomalies and COADS monthly climatology were summed to obtain surface air parameters. Climatologies of downward short wave (COADS) and downward long wave (NCEP) heat fluxes were used. The forcing characteristics of the different simulations are summarized in Table 1.

### Ocean reanalyses

Different ocean reanalyses were used to compute the near-equatorial eastward flux associated with the EUC, pSSCC and sSSCC over the 1970–2008 time period:

- SODAv2.1.6 is forced with the ERA-40 reanalysis. The ocean model is based on the Parallel Ocean Program (POP) model physics with an average 0.25° × 0.4° and 40 vertical levels. The observations that are assimilated include virtually all available hydrographic profile and ocean station data, moored temperature and salinity time series, surface temperature and salinity observations of various types, and nighttime infrared satellite SST data. Model output is mapped onto a uniform 0.5° × 0.5° grid. The analyzed time period is 1958–2008. Model output was downloaded from http://apdrc.soest.hawaii.edu/dods/public_data/SODA/soda_pop2.1.6.
- SODAv2.2.4 is based on the same ocean model and assimilation scheme as v2.1.6 but integrated over a longer period (1871–2008). The ocean model is forced with the Twentieth Century version 2 (20Crv2) reanalysis, which assimilates surface synoptic pressure only. The analyzed time period is 1958–2008. Model output was downloaded from http://apdrc.soest.hawaii.edu/dods/public_data/SODA/soda_pop2.1.6.
- ORAS4 is forced by the ERA-40 reanalysis (as SODAv2.1.6). It is based on the NEMOv3.0 ocean general circulation model. The horizontal grid has 1° × 1° resolution (0.3° meridional resolution in the tropics). Temperature and salinity profiles and along‐track altimeter‐derived sea‐level anomalies are assimilated. The analyzed time period is 1958–2008. Model output was downloaded from https://www.ecmwf.int/en/research/climate-reanalysis/ocean-reanalysis.
- GECCO2 is based on the MITgcm at 1° × 1° (1/3° meridional resolution in the tropics) and 50 levels in the vertical. It is forced by the NCEP RA1 reanalysis (1948–2011). Temperature and salinity profiles, SST and along-track altimeter data area assimilated. The analyzed time period is 1958–2008. Model output was downloaded from https://icdc.cen.uni-hamburg.de/en/gecco2.html.

Flux trends were also computed over more recent time periods (1993–2017 and 1999–2011, see “Discussion”) using 5 different ocean reanalyses provided by the Copernicus Marine Service (https://marine.copernicus.eu/) over 1993–2017: GLORYS2v4 and GLORYS12v1 from Mercator Ocean International, ORAS5 from ECMWF, GloSea5 from the Met Office and C-GLORS05 from the Euro-Mediterranean Center on Climate Change (CMCC). These reanalyses are constrained by the sea level satellite altimetric data from 1993 onward and various sets of in situ observations.

### Linear trend computation

Linear trends of the annual time series were computed using a least-squares method. Statistical significance is presented as a 90% confidence interval, based on a bootstrap method: we computed a 10,000-member synthetic distribution derived by randomly removing data in the annual series. The confidence limits of the trends are indicated by red horizontal bars in the figures and confidence intervals are indicated in Tables 1, 2 and S2.

### Biogeochemical trends

The different terms of the DO evolution were stored for each grid point and each day and averaged for each year. These terms include the total DO rate, the rates due to DO advection, DO vertical diffusion (explicit horizontal diffusion is set to zero in our simulation) and DO air-sea flux. The sum of DO biogeochemical source (production) and sink (consumption) terms (here after O2_sms) were computed as a residual from the difference between the DO rate and the physical terms, as the distinct biogeochemical source and sink terms were not saved during the course of the simulation.

## Supplementary Information

Supplementary Information.

## References

[1] Helm KP, Bindoff NL, Church JA (2011). Observed decreases in oxygen content of the global ocean. Geophys. Res. Lett..

[2] Schmidtko S, Stramma L, Visbeck M (2017). Decline in global oceanic oxygen content during the past five decades. Nature.

[3] Breitburg D (2018). Declining oxygen in the global ocean and coastal waters. Science.

[4] Oschlies A, Brandt P, Stramma L, Schmidtko S (2018). Drivers and mechanisms of ocean deoxygenation. Nat. Geosci..

[5] Bopp L (2013). Multiple stressors of ocean ecosystems in the 21st century: projections with CMIP5 models. Biogeosciences.

[6] Paulmier A, Ruiz-Pino D (2009). Oxygen minimum zones (OMZs) in the modern ocean. Prog. Oceanogr..

[7] Chavez F, Bertrand A, Guevara-Carrasco R, Soler P, Csirke J (2008). The northern Humboldt Current System: brief history, present status and a view towards the future. Prog. Oceanogr..

[8] Fuenzalida R, Schneider W, Garcés-Vargas J, Bravo L, Lange C (2009). Vertical and horizontal extension of the oxygen minimum zone in the eastern South Pacific Ocean. Deep Sea Res. Part.

[9] Espinoza-Morriberón D (2019). Oxygen variability during ENSO in the tropical South Eastern Pacific. Front. Mar. Sci..

[10] Graco M (2017). The OMZ and nutrients features as a signature of interannual and low frequency variability off the Peruvian upwelling system. Biogeosciences.

[11] Luyten JR, Pedlosky J, Stommel H (1983). The ventilated thermocline. J. Phys. Oceanogr..

[12] Czeschel R (2011). Middepth circulation of the eastern tropical South Pacific and its link to the oxygen minimum zone. J. Geophys. Res..

[13] Vergara O (2016). Seasonal variability of the oxygen minimum zone off Peru in a high-resolution regional coupled model. Biogeosciences.

[14] Montes I (2014). High-resolution modeling of the Eastern Tropical Pacific oxygen minimum zone: Sensitivity to the tropical oceanic circulation. J. Geophys. Res. Oceans.

[15] Stramma L (2020). Trends and decadal oscillations of oxygen and nutrients at 50 to 300 m depth in the equatorial and North Pacific. Biogeosciences.

[16] Ito T, Minobe S, Long MC, Deutsch C (2017). Upper ocean O2 trends: 1958–2015. Geophys. Res. Lett..

[17] Bertrand A (2011). Oxygen: A fundamental property regulating pelagic ecosystem structure in the coastal southeastern tropical Pacific. PLoS ONE.

[18] Henley BJ (2015). A tripole index for the interdecadal Pacific oscillation. Clim. Dyn..

[19] Cardich J (2019). Multidecadal changes in marine subsurface oxygenation off central Peru during the last ca. 170 years. Front. Mar. Sci..

[20] Chaigneau A (2013). Near-coastal circulation in the northern Humboldt Current System from shipboard ADCP data. J. Geophys. Res..

[21] Czeschel R, Stramma L, Weller RA, Fischer T (2015). Circulation, eddies, oxygen, and nutrient changes in the Eastern Tropical South Pacific Ocean. Ocean Sci..

[22] Montes I, Colas F, Capet X, Schneider W (2010). On the pathways of the equatorial subsurface currents in the eastern equatorial Pacific and their contributions to the Peru-Chile Undercurrent. J. Geophys. Res. Oceans.

[23] Montes I, Schneider W, Colas F, Blanke B, Echevin V (2011). Subsurface connections in the eastern tropical Pacific during La Niña 1999–2001 and El Niño 2002–2003. J. Geophys. Res. Oceans.

[24] Deutsch C (2014). Centennial changes in North Pacific anoxia linked to tropical trade winds. Science.

[25] Kalvelage T (2013). Nitrogen cycling driven by organic matter export in the south Pacific oxygen minimum zone. Nat. Geosci..

[26] Yang S, Gruber N, Long MC, Vogt M (2017). ENSO driven variability of denitrification and suboxia in the Eastern Tropical Pacific ocean. Glob. Biogeochem. Cycles.

[27] Shigemitsu M, Yamamoto A, Oka A, Yamanaka Y (2017). One possible uncertainty in CMIP5 projections of low-oxygen water volume in the Eastern Tropical Pacific. Glob. Biogeochem. Cycles.

[28] Busecke JJM, Resplandy L, Dunne JPP (2019). The Equatorial Undercurrent and the oxygen minimum zone in the Pacific. Geophys. Res. Lett..

[29] Echevin V (2020). Physical and biogeochemical impacts of RCP8.5 scenario in the Peru upwelling system. Biogeosciences.

[30] Duteil O, Oschlies A, Böning CW (2018). Pacific Decadal Oscillation and recent oxygen decline in the eastern tropical Pacific Ocean. Biogeosciences.

[31] McPhaden MJ, Zhang D (2002). Slowdown of the meridional overturning circulation in the upper Pacific Ocean. Nature.

[32] Drenkard EJ, Karnauskas KB (2014). Strengthening of the Pacific Equatorial undercurrent in the SODA reanalysis: Mechanisms, ocean dynamics, and implications. J. Clim..

[33] Duteil O, Böning CW, Oschlies A (2014). Variability in subtropical-tropical cells drives oxygen levels in the tropical Pacific Ocean. Geophys. Res. Lett..

[34] Czeschel R, Stramma L, Johnson GC (2012). Oxygen decreases and variability in the eastern equatorial Pacific. J. Geophys. Res..

[35] Cheresh J, Fiechter J (2020). Physical and biogeochemical drivers of alongshore pH and oxygen variability in the California Current System. Geophys. Res. Lett..

[36] Sohn BJ, Yeh SW, Schmetz J, Song HJ (2013). Observational evidences of Walker circulation change over the last 30 years contrasting with GCM results. Clim. Dyn..

[37] Pizarro-Koch M (2019). Seasonal variability of the southern tip of the Oxygen Minimum Zone in the eastern South Pacific (30°–38°S): A modeling study. J. Geophys. Res. Oceans.

[38] Srain B (2015). Interdecadal changes in intensity of the oxygen minimum zone off Concepción, Chile (∼36°S), over the last century. Biogeosciences.

[39] Bograd SJ (2008). Oxygen declines and the shoaling of the hypoxic boundary in the California Current. Geophys. Res. Lett..

[40] Deutsch C, Brix H, Ito T, Frenzel H, Thompson L (2011). Climate-forced variability of ocean hypoxia. Science.

[41] Brandt P (2021). Atlantic Equatorial Undercurrent intensification counteracts warming-induced deoxygenation. Nat. Geosci..

[42] Carrit DE, Carpenter JH (1966). Recommendation procedure for Winkler analyses of sea water for dissolved oxygen. J. Mar. Res..

[43] Shchepetkin AF, McWilliams JC (2005). The regional oceanic modeling system: A split-explicit, free-surface, topography-following-coordinate ocean model. Ocean Model.

[44] Aumont O, Ethé C, Tagliabue A, Bopp L, Gehlen M (2015). PISCES-v2: An ocean biogeochemical model for carbon and ecosystem studies. Geosci. Model Dev..

[45] Espinoza-Morriberón D (2017). Impacts of El Niño events on the Peruvian upwelling system productivity. J. Geophys. Res. Oceans.

[46] Key RM (2004). A global ocean carbon climatology: Results from Global Data Analysis Project (GLODAP). Glob. Biogeochem. Cycles.

[47] Goubanova K (2011). Statistical downscaling of sea-surface wind over the Peru–Chile upwelling region: diagnosing the impact of climate change from the IPSL-CM4 model. Clim. Dyn..

[48] Cambon G (2013). Assessing the impact of downscaled winds on a regional ocean model simulation of the Humboldt system. Ocean Model.

