# Supplementary material for: Evidences and drivers of ocean deoxygenation off Peru over recent past decades
Source: Sci Rep. 2021 Oct 13;11:20292. doi: 10.1038/s41598-021-99876-8 (PMC8514543; doi:10.1038/s41598-021-99876-8)
Supplement: Supplementary file 1 — Supplementary Information. [file 41598_2021_99876_MOESM1_ESM.pdf]

Supplementary Information: Evidences and drivers of the deoxygenation off Peru over recent past decades

Espinoza-Morriberón D.[1], V. Echevin[2], D. Gutiérrez[1,3], J. Tam[1], M. Graco[1], J. Ledesma[1], F. Colas[2]

[1]Instituto del Mar del Peru (IMARPE), Esquina general Gamarra y Valle, Callao, Peru.

[2]LOCEAN-IPSL, IRD/Sorbonne Université/CNRS/MNHN, Paris, France.

[3]Programa de Maestría en Ciencias del Mar, Universidad Peruana Cayetano Heredia, Lima, Perú

1. Computation of Dissolved Oxygen (DO) consumption using linear trends

In order to interpret the role of the biogeochemical (BGC) oxygen consumption in the the oxygen depletion observed during the last decades, we analyze the rates at 70 m depth, where the maximum deoxygenation off Peru is found. More details about the oxygen equations in the PISCES model are described in Espinoza-Morriberón et al.[9]. DO consumption by BGC processes decreases by ~ 0.3 μmol kg^{-1} year^{-2} at 70 m depth (see Fig. S5b). If we model this O2_sms (biogeochemical sources minus sinks, computed from the trends: O2_sms=O2_rate - physical terms) change by a linear trend, this leads to:

$$O2_{sms}(t) = b.t$$

With t = time in year and b = 0.3 μmol kg^{-1}year^{-2}. The change of DO due to BGC processes is thus:

$$\frac{dO2_{BGC}}{dt} = O2_{sms}(t) = b.t$$

and the integration over 40 years leads to:

$$\Delta O2_{BGC} = O2_{BGC}(2008) - O2_{BGC}(1970) = \int (b.t)dt = \frac{1}{2}b.[39years]^2$$
$$= +230\mu molkg^{-1}$$

The oxygen trend due to both physical and biogeochemical processes at 70 m is ~-10 μmol $kg^{-1}dec^{-1}$ (see Fig. 1c) which corresponds to an oxygen change over 4 decades of:

$$\Delta O2_{tot} = \Delta O2_{BGC} + \Delta O2_{PHYS} = -10 * 4 = -40\mu molkg^{-1}$$

The DO change at 70 m depth due to the physical trend is thus:

$$\Delta O2_{PHYS} = \Delta O2_{tot} - \Delta O2_{BGC} = -270\mu molkg^{-1}$$

This demonstrates that the physical processes largely drive the DO loss off nearshore Peru (6°S-16°S, 0-200 km to the coast) whereas the BGC processes partly compensate it.

2. Supplementary Figures and Tables

This supplementary information provides figures and tables in order to support the evidence of deoxygenation off Peru.

Fig. S1 shows the oxycline depth linear trend between 1970 and 2008 in the Si-control simulation. The shoaling of the oxycline occurs in almost the whole domain of study. The deoxygenation is strongest in the north of Peru, related to the influence of the eastward oxygen-rich subsurface zonal currents.

Fig. S2 indicates that nearshore shoaling of the oxycline is observed only from the 70s to the 2000s. From 1958 to 1970, the oxycline deepened, indicating a thickening of the surface oxygenated layer. Both model output and IMARPE data show the shift from oxygenated (1958-1970) to deoxygenated (1970-2000s) conditions. **Fig. S2b** indicates that the stronger nearshore oxycline shoaling trends is observed from the 1970s to the early 1990s.

Fig. S3 shows a vertical section of the Si-control simulation mean zonal velocity at 88°W. The dashed boxes are used to compute the eastward mass and oxygen fluxes associated to the different subsurface currents (EUC, pSSCC and sSSCC). These subsurface currents transport relatively oxygenated waters towards the coast over a large depth range (~50-400 m depth). It is noteworthy that the equatorial subsurface currents are tightly correlated with the oxycline depth off Peru, in particular the EUC and pSSCC with the latitude band of 6°S-8°S and the sSSCC with the latitude band of 8°S-10°S (see **Table S1**).

The role of the oxygen consumption by BGC processes is also illustrated in **Fig. S4**. This figure shows the nearshore-average profile of oxygen consumption by BGC processes, with a maximum rate at ~70 m depth (~-68 µmol kg^{-1} $year^{-1}$). The trends of oxygen consumption is positive so oxygen consumption due to remineralization of organic matter decreases in the study period, possibly due to a reduced oxygen availability. This analysis and the estimation shown previously (in section 1 of supplementary information) demonstrate that physical processes are the main drivers of the modelled deoxygenation.

Fig. S5 shows the zonal velocity associated to the EUC and SSCCs at 100°W in four different ocean reanalyses. GECCO2 and ORAS4 do not represent correctly the SSCCs. The section used to compute the eastward mass flux driven by the currents is indicated. The decrease of the associated eastward fluxes observed in three of the four reananalyses is consistent with the regional control simulation (Si-control) (see **Fig. 4**).

In addition, assuming that the eastward mass flux is the main driver of the nearshore DO trends, we investigate the recent eastward mass flux trend (until 2017) of five recent ocean reanalyses in order to elucidate whether deoxygenation may be going on off Peru. **Fig. S6** and **Table S2** show the time series and trends of the eastward mass flux, respectively, for different time periods. A deoxygenation is statistically evidenced in three reanalyses between 1993-2008. However, in recent years (2005-2017), a statistically significant strengthening of the eastward flux is found in two out of five products. This increase is consistent with the oxygenation observed in recent years at 12°S off Peru [10].

Last, the number and spatial distribution of IMARPE DO measurements used to compute the DO trends are shown in **Fig. S7** and **Table S3**. The sampling was clearly intensified during the recent decades. However, it is not homogeneous alongshore: the area northward of 13°S is better sampled during the period of study (1970-2008). Besides, there is more data (~60 %) collected close to the coast (0-100 km) than between 100 and 200 km from the coast (~40%).

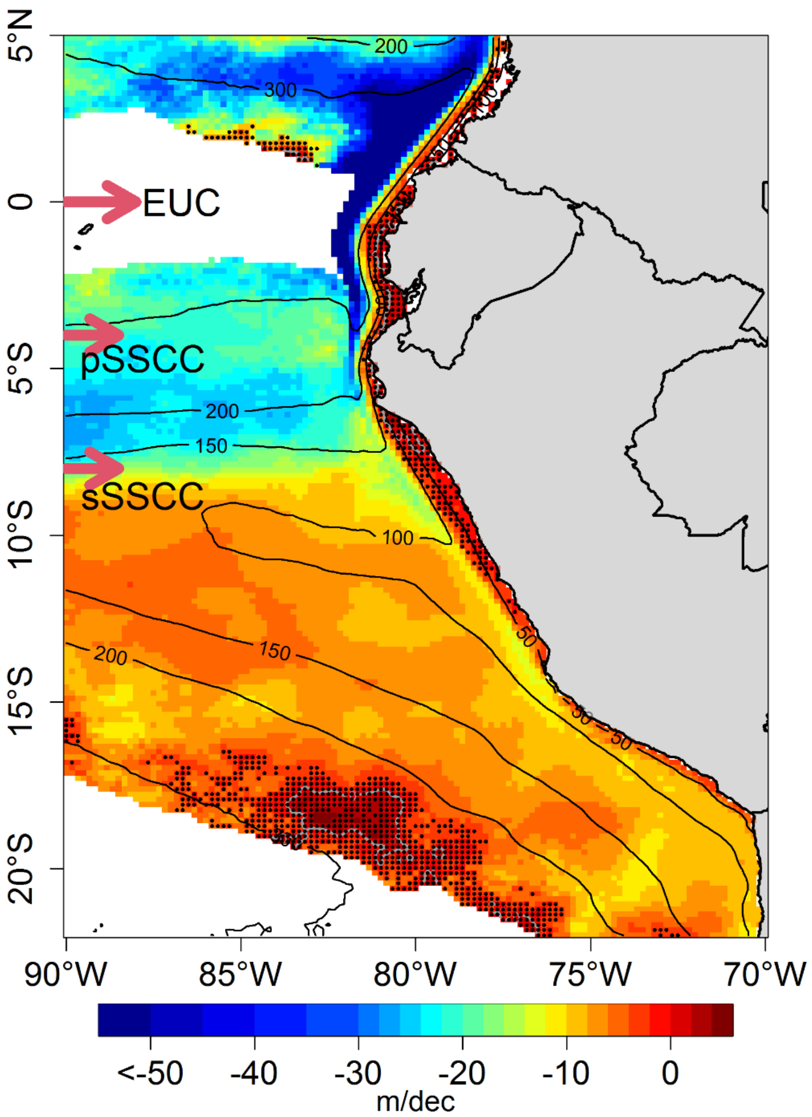

Figure S1. Trend of the oxycline depth (in m dec^{-1}) computed between 1970 and 2008 from the Si-control simulation (**Table 1**). Negative and positive values indicate a shoaling and deepening of the oxycline depth, respectively. Black contours represent the mean oxycline depth and gray contours indicate where trends are positive (a shoaling of the oxycline is found in most of the region). Regions with non statistically significant trend are indicated by black dots. Position of the EUC, jet 1 (pSSCC) and jet2 (sSSCC) are indicated by red arrows. The figure was generated using the software R (R Core Team, version 4.1.1., http://www.R-project.org/).

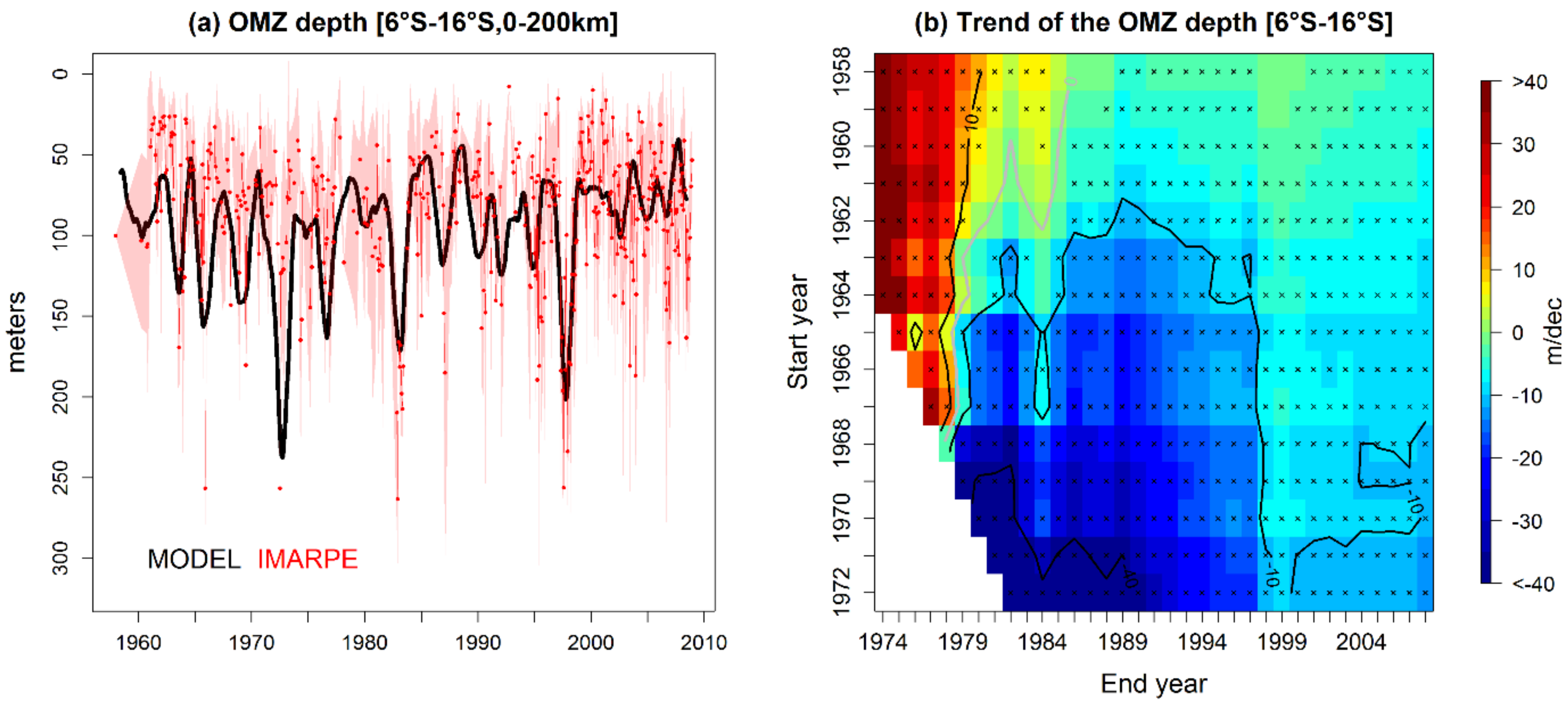

Figura S2. (a) Oxycline depth from model (black line, Si-control simulation) and IMARPE (red line and points) from 1958 to 2008, computed in a coastal band (6°S-16°S, 0-200 km from the coast). The red envelop represents the confidence intervals of IMARPE data. (b) Linear trend of the oxycline depth (colors and contours, in m dec^{-1}) from the Si-control simulation. The trend is computed in the same coastal band as in (a), but for different time periods (start year, end year) longer than 10 years. Black dots represent statistically significant trends. The figures were generated using the software R (R Core Team, version 4.1.1., http://www.R-project.org/).

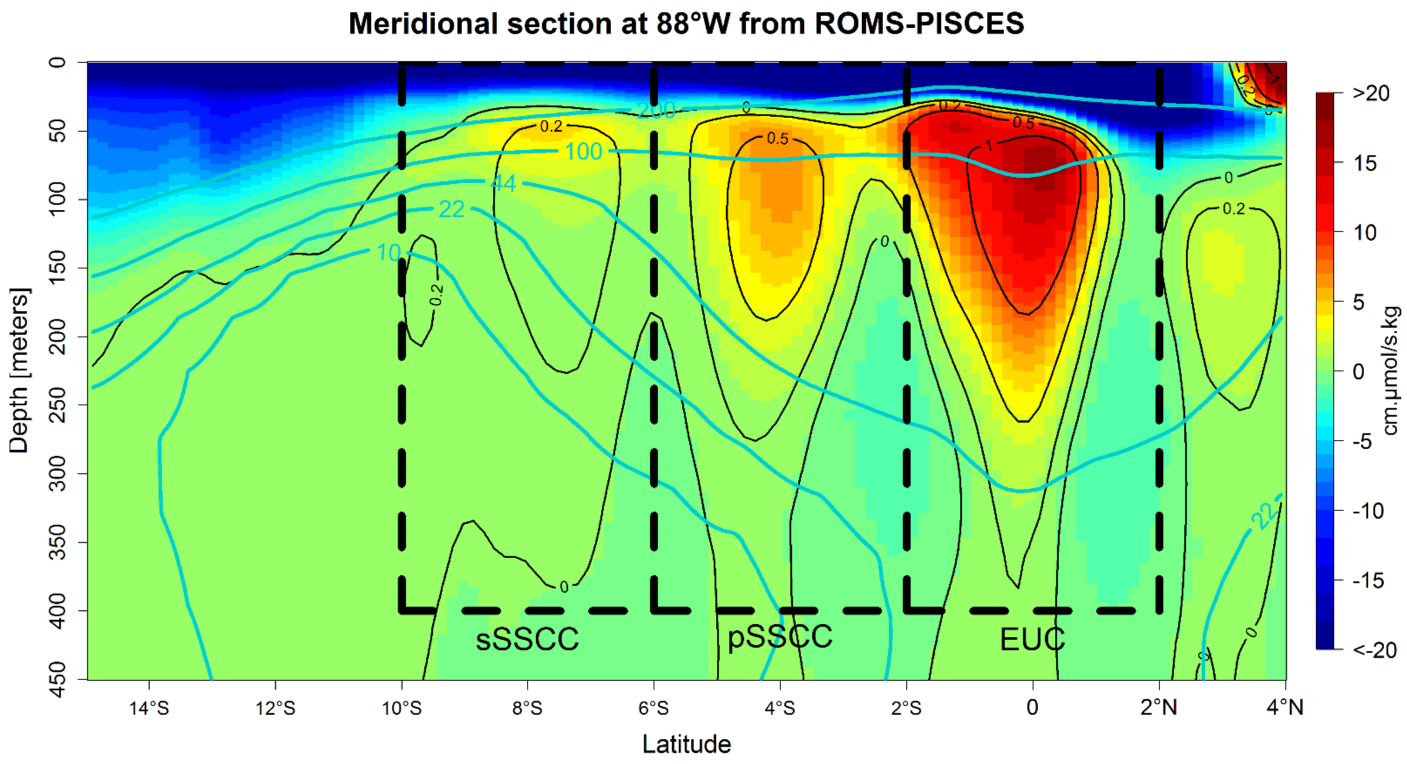

Figure S3. Meridional section (88°W) of mean eastward oxygen flux (color, in cm.µmol $kg^{-1}.s^{-1}$) from the Si-control simulation for 1970-2008. Black and blue contours represent zonal currents (in cm s^{-1}) and oxygen concentration (in µmol kg^{-1}), respectively. Black boxes represent the sections used to compute the eastward oxygen flux, mass flux and oxygen trends from the EUC and jets (pSSCC and sSSCC). Eastward fluxes are computed for eastward currents larger than 0.2 cm s^{-1} (Table 2). The figure was generated using the software R (R Core Team, version 4.1.1., http://www.R-project.org/).

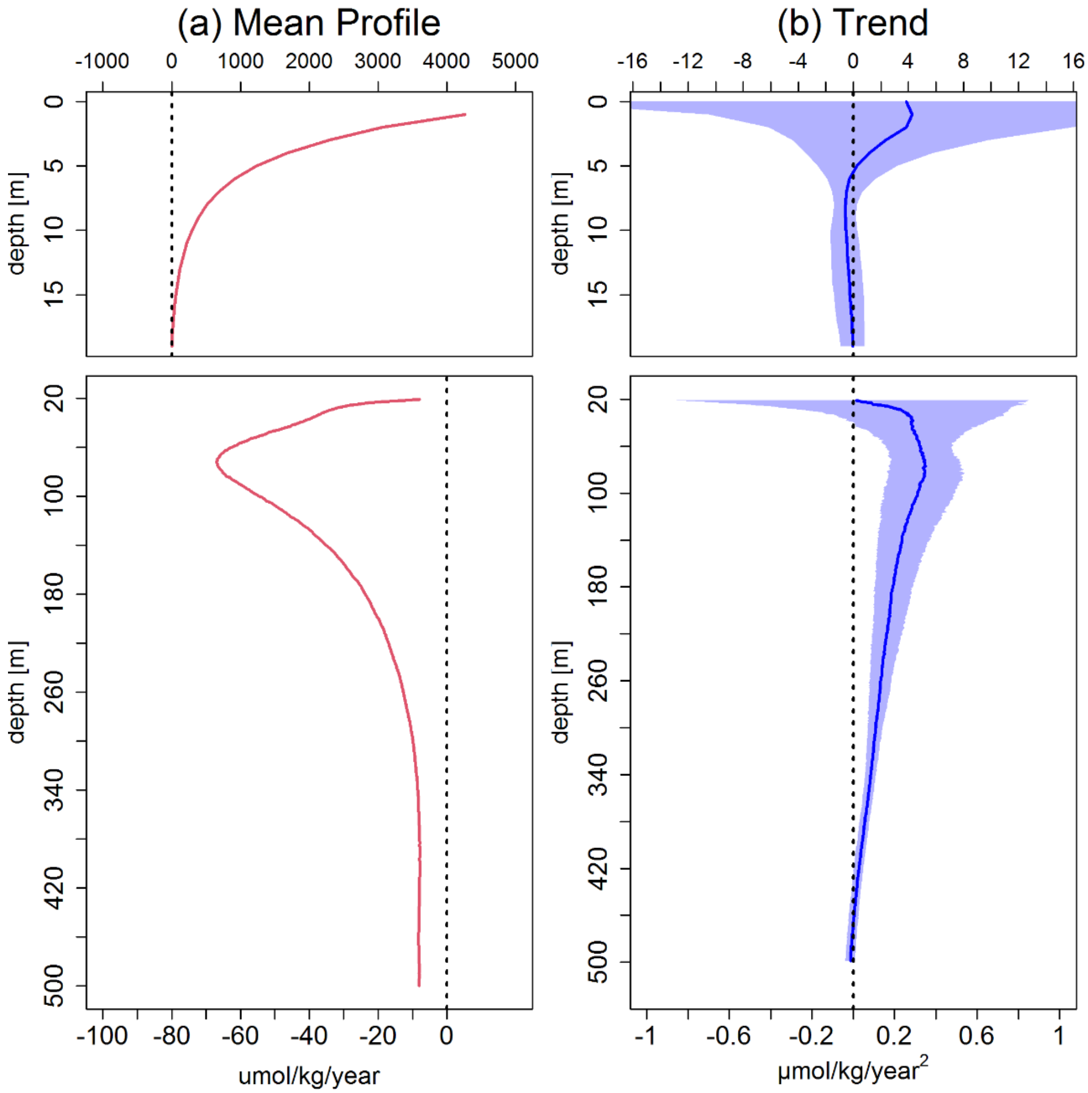

Figure S4. (a) O_2 sms (DO biogeochemical sources minus sinks) averaged in a coastal band (200 km, 6°S-16°S) for 1970-2008. Negative values in (a) indicate that o2_sms is consuming oxygen, *e.g.* at a maximum rate of 70 μmol kg^{-1} $year^{-1}$ near 70 m depth. (b) O_2 sms trend (in μmol kg^{-1} $year^{-2}$) computed in the same coastal band as in (a). Positive trend values indicate a decrease in oxygen consumption by biogeochemical processes. Note the different horizontal scales above and below 20 m in (a) and (b). The figures were generated using the software R (R Core Team, version 4.1.1., http://www.R-project.org/).

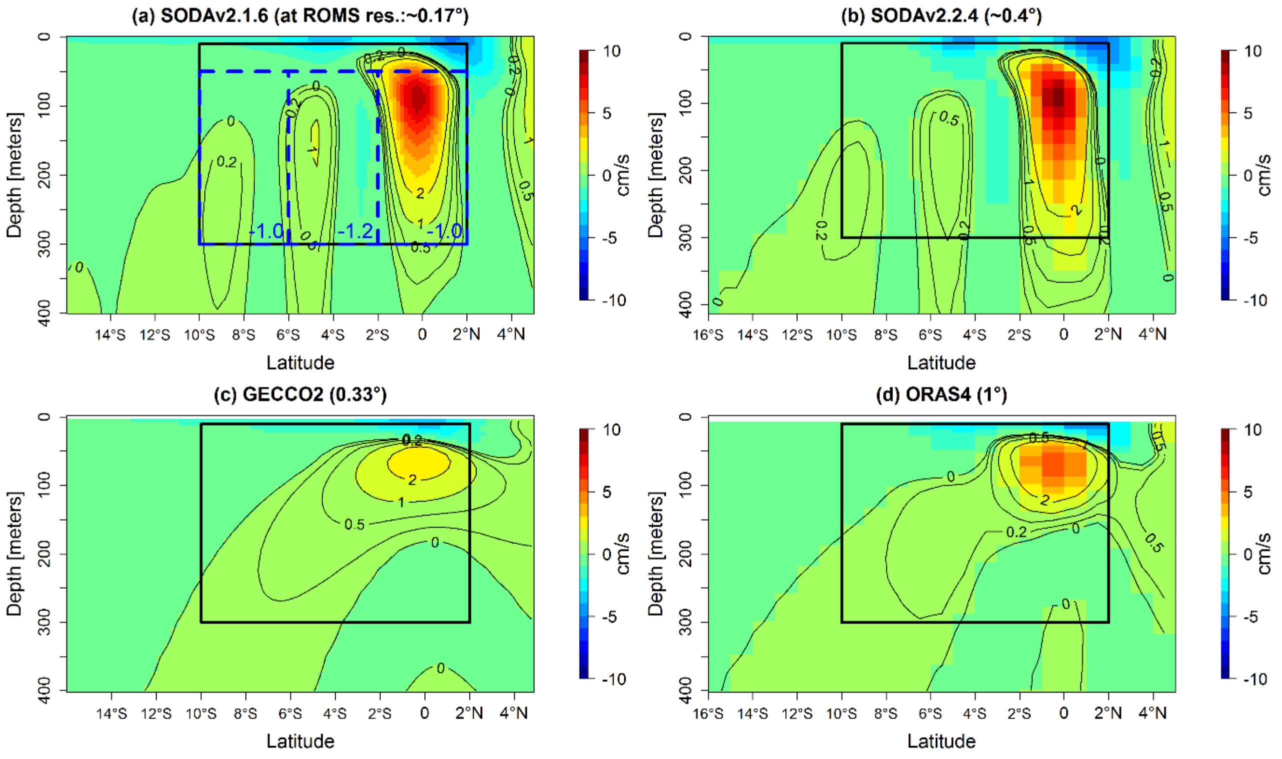

Figure S5. Meridional section at 100°W of mean zonal currents (color and black contours, in cm s^{-1}) from the OGCMs. The black rectangles indicate the area used to compute the total eastward flux (u>0.2 cm s^{-1}) associated with the EUC, pSSCC and sSSCC. Blue boxes in (a) represent the area in which a desoxygenation trend of equatorial waters[21] was added to the climatological OBC (Si-control-DO simulation, Table 1). Numbers in blue font in (a) indicate the oxygen trend (in µmol kg yr^{-1}) derived from [21]. The figures were generated using the software R (R Core Team, version 4.1.1., http://www.R-project.org/).

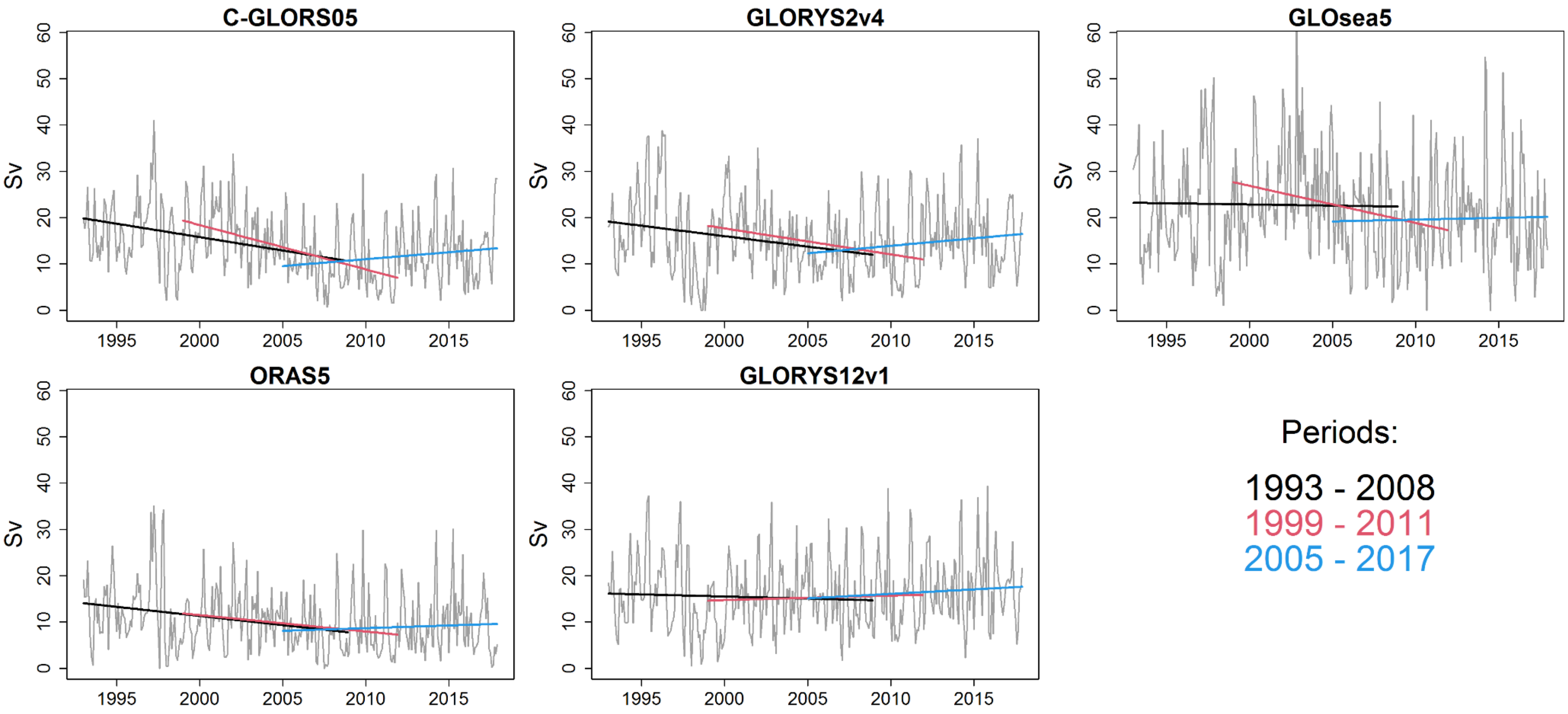

Figure S6. Eastward flux (in Sv) at 100°W associated to the EUC, pSSCC and sSSCC for 5 ocean reanalysis (C-GLORS05, GLORYS2v4, GLOseas5, ORAS5, GLORYS12v1) over 1993-2018. The flux is defined for u>0.2 cm s^{-1}. Linear trends for different time periods (black: 1993-2008, red: 199-2011, blue: 2005-2017) are shown and values are indicated in Table S3. The figures were generated using the software R (R Core Team, version 4.1.1., http://www.R-project.org/).

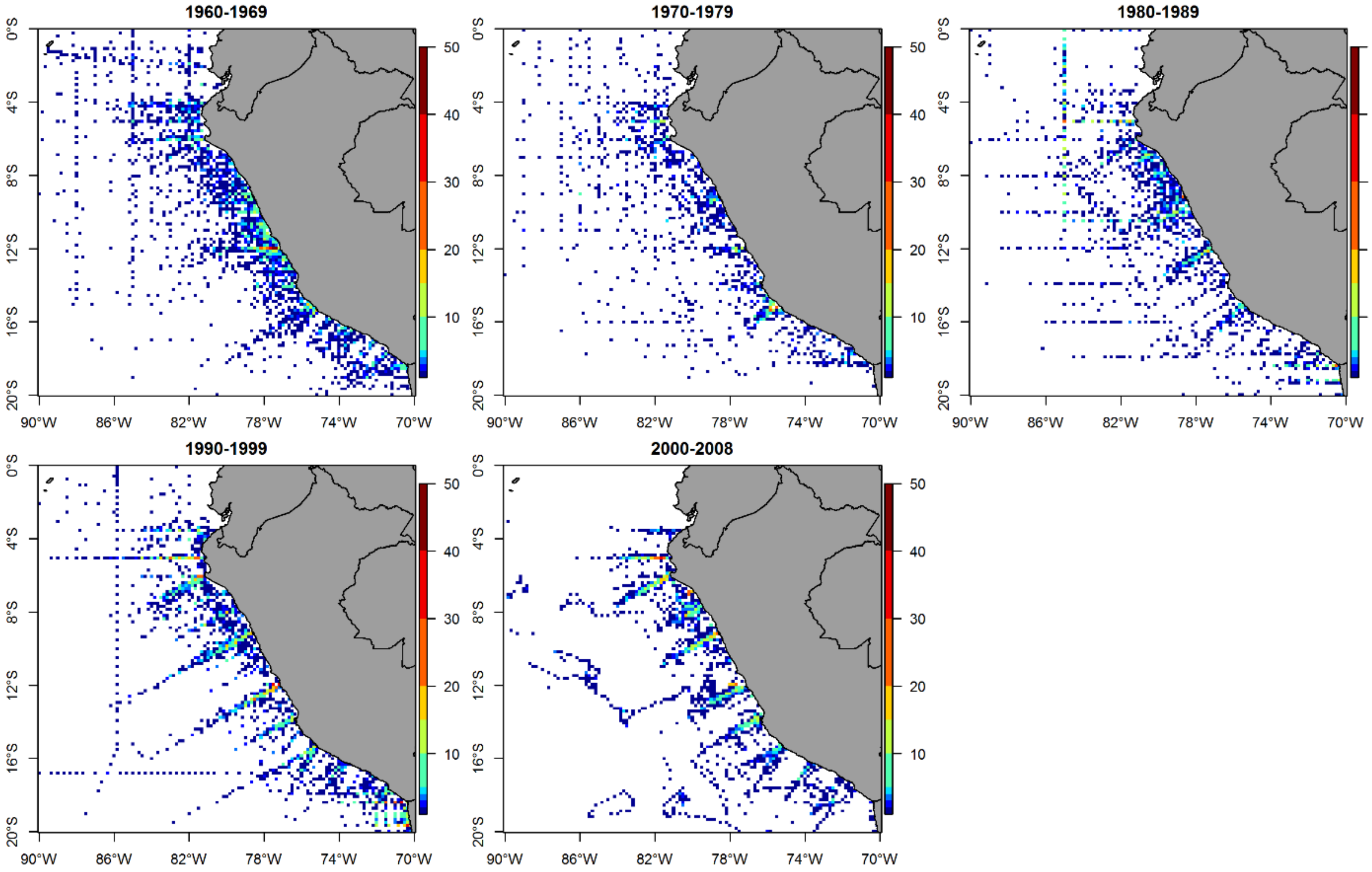

Figure S7. Number of the oxycline depth observations per decade. IMARPE data was gridded onto the model horizontal resolution (1/6°). The figures were generated using the software R (R Core Team, version 4.1.1., http://www.R-project.org/).

Table S1. Correlation between the monthly time series of the DO eastward flux associated with each eastward subsurface currents (EUC, pSSCC and sSSCC) and the nearshore oxycline depth (0-200 km) averaged over different latitude ranges. The time lag (in months) corresponding to the maximum correlation is indicated in parentheses. A negative time lags indicates that the flux leads oxycline depth. Statistically significant correlations are marked in bold font.

	Correlation				
	6°S - 8°S	8°S - 10°S	10°S - 12°S	12°S - 14°S	14°S - 16°S
EUC	**0.55 (0)**	0.49 (0)	0.43 (0)	0.42 (-1)	0.45 (-1)
pSSCC	**0.81 (0)**	0.78 (0)	0.71 (0)	0.63 (0)	0.54 (0)
sSSCC	0.86 (0)	**0.89 (0)**	0.86 (0)	0.79 (0)	0.71 (0)

Table S2. Linear trends (with confidence intervals) by different periods (1993-2008, 1999-2011, 2005-2017) of eastward equatorial currents (EUC+pSSCC+sSSCC) flux, in Sv dec^{-1}, at 100°W for 5 different reanalysis global products: C-GLORS05, GLORYS2v4, GLOseas5, ORAS5, GLORYS12v1. (*) means the trend is statistically significant.

Models	1993 - 2008		1999 - 2011		2005 - 2017	
	Trend (Sv dec^{-1})	Conf. Int.	Trend (Sv dec^{-1})	Conf. Int.	Trend (Sv dec^{-1})	Conf. Int.
C-GLORS05	-5.76*	[-7.39 ; -4.24]	-9.57*	[-11.87 ; -7.15]	2.99*	[0.66 ; 5.31]
GLORYS2v4	-4.51*	[-6.41 ; -2.59]	-5.59*	[-8.18 ; -3.00]	3.28*	[0.93 ; 5.51]
GLOsea5	-0.51	[-3.35 ; 2.24]	-7.94*	[-11.97 ; -4.50]	0.63	[-2.64 ; 4.76]
ORAS5	-3.95*	[-5.54 ; -2.33]	-3.53*	[-5.67 ; -1.20]	1.17	[-0.88 ; 3.18]
GLORYS12v1	-0.88	[-2.86 ; 0.95]	0.92	[-1.49 ; 3.73]	1.97	[-0.62 ; 4.40]

Table S3. Number of the oxycline depth observations computed from IMARPE data at the horizontal resolution of the simulation (1/6°) by decades, latitude bands and coastal (0-100 km) and oceanic (100–200 km) zones. The percentages of data in the coastal and oceanic band are computed by decade. In adittion, the number of months with data are shown in the gray boxes, and the percentages represent the relative amount of moth with data by decade respect to the total months with data between 1970 and 2008.

	[4°S-7°S]				[7°S-10°S]				[10°S-13°S]				[13°S-16°S]			
	[0-100km]	[100-200km]	[0-200km]	N° months with data	[0-100km]	[100-200km]	[0-200km]	N° months with data	[0-100km]	[100-200km]	[0-200km]	N° months with data	[0-100km]	[100-200km]	[0-200km]	N° months with data
[1970-1979]	93 (56%)	73 (44%)	166	36 (18%)	91 (58%)	65 (42%)	156	33 (16%)	111 (69%)	49 (31%)	160	41 (17%)	154 (71%)	62 (29%)	216	31 (22%)
[1980-1990]	131 (66%)	69 (34%)	200	32 (16%)	186 (63%)	108 (37%)	294	41 (20%)	131 (48%)	144 (52%)	275	49 (20%)	76 (66%)	39 (34%)	115	27 (19%)
[1990-1999]	224 (61%)	145 (39%)	369	61 (31%)	187 (48%)	199 (52%)	386	63 (30%)	262 (66%)	138 (34%)	400	53 (21%)	160 (66%)	82 (34%)	242	37 (27%)
[2000-2008]	283 (64%)	162 (36%)	445	71 (35%)	306 (60%)	203 (40%)	509	70 (34%)	401 (75%)	133 (25%)	534	103 (48%)	163 (67%)	82 (33%)	245	44 (32%)
[1970-2008]	731 (62%)	449 (38%)	1180	200	770 (57%)	575 (43%)	1345	207	905 (66%)	464 (34%)	1369	246	553 (68%)	265 (32%)	818	139
